# Expansion of the Beta-Proteobacterial Genus *Ca*. Ichthyocystis: A Case Report of Epitheliocystis in the Pompano *Trachinotus ovatus*

**DOI:** 10.3390/pathogens11040421

**Published:** 2022-03-30

**Authors:** Maria Chiara Cascarano, Pantelis Katharios

**Affiliations:** 1 Department of Biology, School of Sciences and Engineering, University of Crete, 70013 Heraklion, Greece; mariachiaracascarano@gmail.com; 2 Institute of Marine Biology, Biotechnology & Aquaculture (IMMBC), Hellenic Centre for Marine Research (HCMR), 71500 Heraklion, Greece

**Keywords:** beta-proteobacteria, intracellular pathogens, gills, Mediterranean Sea, Carangidae

## Abstract

Epitheliocystis is a disease caused by a wide variety of host-specific intracellular bacteria infecting fish gills. In the Mediterranean Sea, epitheliocystis has been recently associated with a novel genus of beta-proteobacteria, the *Ca*. Ichthyocystis genus. In the present study, we report a case of epitheliocystis in a wild-caught specimen of pompano *Trachinotus ovatus* in Crete, Greece. Molecular analysis of partial 16s rRNA sequence led to the discovery of a putative novel species of the *Ca*. Ichthyocystis genus. Investigation of the phylogenetic relationship between closely related sequences deposited in NCBI suggests that bacterial ancestors in gilthead seabream might have a pivotal role in the differentiation of genus.

## 1. Introduction

Epitheliocystis is a disease caused by intracellular bacteria that infect cells of the skin and respiratory epithelium of fish [1]. It is typically diagnosed by the observation of characteristic rounded inclusions—cysts—that are formed in a progressively hypertrophied host cell during the process of bacterial intracellular replication. 

Since the intracellular nature of the infecting agents complicates the ability of in vitro cultivation, epitheliocystis is only studied during natural outbreaks, and it is closely observed mostly in aquaculture farmed species. In fact, following the first description of the disease in 1920 [2], a number of farmed fish has been reported to be affected by this condition to date, while only a small number of infections have been reported from wild host species (for an extended list of hosts and associated pathogens one might consult Blandford et al., 2018 [1]).

Aetiological agents causing epitheliocystis are host specific and include numerous members of the Chlamydial phylum (reviewed in Stride, Polkinghorne, and Nowak 2014 [3] updated in Blandford et al., 2018 [1]), and fewer but rising in number, proteobacteria including gamma-proteobacteria from the *Endozoicomonas* genus [4,5] and beta-proteobacteria [6,7,8,9].

Beta-proteobacteria are emerging as principal causative agents of mortalities in epitheliocystis mixed infections [6]. This phylum is receiving increasing attention since one of its members, the uncultured bacterium *Ca*. Branchiomonas cysticola [9], has been recently recognized as a major contributor to the complex gill disease [10], a multifactorial disease responsible for important economic losses in the Atlantic salmon aquaculture industry [11]. Epitheliocystis-related beta-proteobacteria include *Ca*. Branchiomonas cysticola, infecting salmonids in Norway, Ireland, Canada, and Chile [7,9,12,13,14,15], the clone BK-BJC, identified in epitheliocystis in the lake trout, *Salvelinus namaycush* in Canada [8], and *Ca*. Ichthyocystis affecting reared gilthead seabream *Sparus aurata* [6], and the greater amberjack *Seriola dumerili* [16] in the Mediterranean Sea. The *Ca*. Ichthyocystis genus currently includes only two species, *Ca*. Ichthyocystis hellenicum and *Ca*. Ichthyocystis sparus, coinfecting gilthead seabream in different farming areas of Greece [6] and a third putative agent in farmed greater amberjack [16]. The genus shares a common ancestor with the aforementioned clone BK-BJC and *Ca*. Branchiomonas cysticola and is of critical importance because currently, it comprises the only epitheliocystis-related beta-proteobacteria fully sequenced [6,17]. Their genomic analysis has, in fact, revealed a wide range of virulence factors and host-associated features [17], shedding light on the mechanisms that drive infection in epitheliocystis.

While most epitheliocystis cases are described in aquaculture fish species, as discussed earlier, uncultured wild-caught fish represent a rare excellent opportunity to explore the real extent of the environmental diversity of the agents causing this disease. In the present case report, we identified a putative novel species of the genus *Ca*. Ichthyocystis in a wild-caught pompano *Trachinotus ovatus,* (Linnaeus, 1758) (fam. Carangidae), individual from Crete, Greece, hosted in Cretaquarium, the public aquarium of the Hellenic Centre for Marine Research (HCMR). The accidental findings of epitheliocystis combined with molecular data indicate that beta-proteobacteria from the *Ca*. Ichthyocystis genus have a wider host range and can potentially infect not only sparid but also carangid fish. The phylogenetic relationship between agents on different hosts is discussed to shape hypotheses on factors driving the evolution of the genus.

## 2. Materials and Methods

In January 2020, a case of mortality of wild-caught pompano, *Trachinotus ovatus,* was reported in the Cretaquarium, a public aquarium of the Hellenic Centre for Marine Research (HCMR) in Gournes, Crete, Greece, in a tank dedicated to this fish species (single-species tank). To determine potential infectious causes of mortality in the tank, a freshly dead juvenile (9 cm in length) was brought to the microbiology laboratory of the Institute of Marine Biology, Biotechnology, and Aquaculture of the Hellenic Centre for Marine Research. The specimen was subjected to routine screening, including visual examination of mucous swabs and gills, dissection, and inspection of internal organs. Additionally, to exclude the possibility of a systemic infection, microbiological sampling of bacteria from the kidney was performed on 2% Tryptic Soy Agar (TSA 2%), and plates were inspected for growth for seven days. Following the observation of sporadic cyst-like structure in the gills (light microscopy), whole gill arches were preserved in 96% ethanol, RNA *later*, and 10% phosphate buffer formalin (PBF). 

PBF preserved gill samples were used for histopathological examination. Fixed tissue was dehydrated in progressively increasing concentrations of ethanol (from 70 to 95%) and subsequently fixed in glycol methacrylate resin (Technovit 7100, Heraeus Kulzer, Germany) [18]. Thin sections of 4 μm were obtained with the use of a microtome (RM 2245 Leica Biosystems, Nussloch, Germany), stained with a polychrome stain (methylene blue/azure II/basic fuchsin) [18], and visualized with a light microscope. 

DNA extraction was performed on the 96% ethanol preserved gill arch, using the DNeasy Blood and Tissue kit (Qiagen inc., Toronto, ON, Canada) following the protocol Purification of Total DNA from Animal Tissues. Extracted DNA was PCR-amplified with primers for known epitheliocystis agents, including *Endozoicomonas* spp. [4] *Ca*. Ichthyocystis genus [6] and Chlamydial genus (16SIG F and 16SB1 primers) [19,20,21]. PCR products were run on 1% agarose gel and visualized with ethidium bromide transillumination. Positive 16S rRNA amplicons were purified using QIAquick PCR Purification Kit (Qiagen), resuspended in ultrapure water, and sequenced using Sanger dideoxy sequencing technology (ABI3730xl). Sequenced chromatograms of both forward and reverse primers were quality inspected and aligned using Geneious 9.1. Consensus sequence was extracted and submitted to the NCBI database under the accession number OM658542.

To explore potential phylogenetic relations of the pathogen with other bacteria, its partial 16S rRNA sequence was blasted against the NCBI nucleotide database, and a selection of representative sequences with more than 90% identity was downloaded and aligned using Muscle v3.8.31 [22]. An alignment file was then used to build a maximum likelihood tree (1000 bootstrap) using MegaX (version 10.1.8) [23].

## 3. Results and Discussion

**Figure 1:**
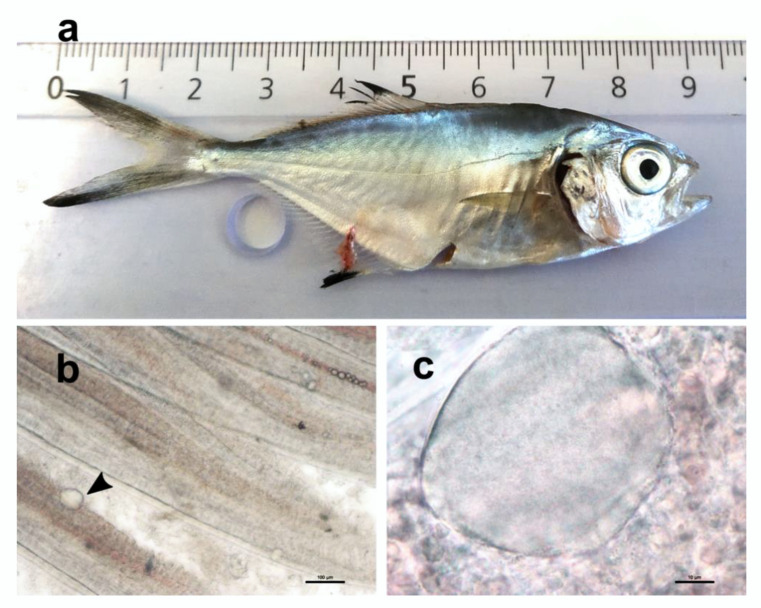
(**a**) Sampled juvenile of pompano *Trachinotus ovatus*. (**b**) Wet mount of gill filaments visualized in the stereoscope showing a cyst-like structure (arrowhead). (**c**) Higher magnification of a cyst displaying granular content.

The sampled specimen did not display any external sign of disease (Figure 1a). Screening of mucous swabs and internal organs, and microbiological sampling of bacteria from the kidney, did not show the presence of pathogens. It was, therefore, suggested that unidentified factors other than infection or parasitism were causing the observed mortalities more likely connected to adaptation stress to captivity.

Wet mount of gill filaments indicated the infrequent presence of cyst-like structures with granular content (Figure 1b,c).

**Figure 2:**
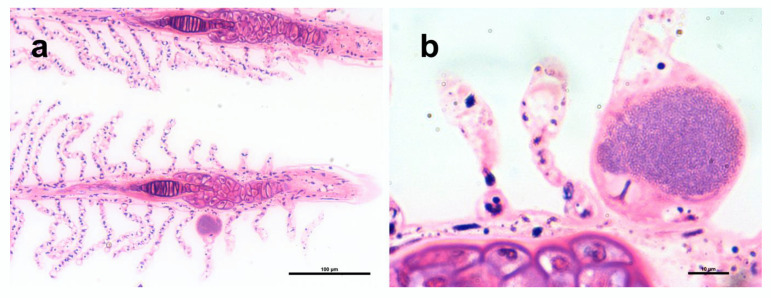
Histopathology of *Trachinotus ovatus* gills, polychrome stain. (**a**) Gill filaments with a cyst on the lamella. (**b**) Higher magnification of a cyst with a characteristic granular texture.

Epitheliocystis was confirmed through histology, with low intensity of infection of approximately 3 cysts in 30 filaments. Cysts were located on the secondary lamellae (Figure 2a,b) and were not associated with proliferative response, inflammation, and lesions. Inclusions had an approximate diameter of 50 μm and displayed a basophilic granular content (Figure 2b).

Mortalities due to epitheliocystis, according to our knowledge, have never been reported in wild fish [24]. Under culture conditions, mortalities are usually associated with severe disruption of the respiratory tissue that likely causes the impairment of its functions. This includes cases in which high intensity of infection (estimated by the number of cysts per filament as in Stride and Nowak 2014 [25]) and extensive signs of host response (such as epithelial proliferation or fusion of lamellae) are observed (reviewed in [24]). 

Contrarily to what is shown in other studies, where the high intensity of infection has been clearly related to mortalities [26], the low infection intensity observed here, together with the absence of epithelial proliferation or disruption of the gill epithelium, indicates that it is highly unlikely that epitheliocystis was the cause of mortality of pompano.

Since a growing number of bacteria and hosts have been associated with epitheliocystis [1] and only a generic report of the disease has been made on the pompano [27], we investigated the case further and attempted to attribute the lesions to a specific agent. Molecular analysis indicated that the gills were negative for chlamydial or *Endozoicomonas* spp. agents and positive for *Ca*. Ichthyocystis. Sequencing of the PCR product obtained with *Ca*. Ichthyocystis primers produced an 870 bp amplicon of high quality.

**Figure 3:**
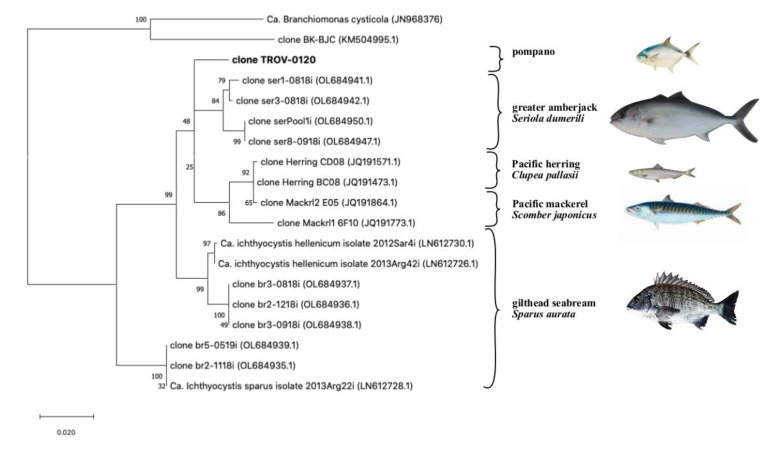
Updated phylogeny of the *Ca*. Ichthyocystis following the sequencing of the partial 16s rRNA gene in pompano (clone TROV-0120, in bolt). Fish hosts from which sequences were obtained are shown on the right; other beta-proteobacteria known to cause epitheliocystis in fish, as clone BK-BJC [8] and *Ca*. Branchiomonas cysticola [9], are included as an outgroup. Highest log-likelihood tree (−4287.45) obtained in MegaX using the Maximum Likelihood method and the Tamura-Nei Model. The tree is drawn in scale using substitutions per site as a measure of branch length; percentage of trees in which sequences cluster together is shown on the branches.

When the partial 16S rRNA sequence was blasted against the NCBI database, we found similarities with uncultured beta-proteobacteria sequences isolated from different fish hosts, including greater amberjack, *Seriola dumerilii* (fam: Carangidae), Pacific mackerel, *Scomber japonicus* (fam. Scombridae), Pacific herring, *Clupea pallasii* (Fam Clupeidae) and gilthead seabream, *Sparus aurata* (fam Sparidae) (Figure 3).

The highest percentage of similarity was found with a cluster of sequences (97.5 to 97.9% identity) retrieved from epitheliocystis in greater amberjack in Crete, Greece [16]. Another group of sequences from Pacific herring and Pacific mackerel had a lower similarity (96.2 to 97.2% identity, 77% query cover). Finally, a similarity was found with sequences from the known *Ca*. Ichthyocystis genus previously identified from epitheliocystis lesions in gilthead seabream [6]. Specifically, the amplicon of the putative novel pathogen had an identity ranging between 95.9–96.1%, with sequences clustering with *Ca*. Ichthyocystis hellenicum and 93.3 to 92.7% with sequences clustering with *Ca*. Ichthyocystis sparus.

Although the full 16S rRNA sequence is required to define a new species, according to what was defined in Yarza et al., 2014 [28], a similarity of 97.9% with the closest relative (AN:OL684941.1) indicate that the bacterium causing epitheliocystis in *Trachinotus ovatus* probably belongs to a novel species of the genus *Ca*. Ichthyocystis. If this is the case, once again, a different bacterial species is observed in a different host, supporting the hypothesis that epitheliocystis agents are indeed host specific [1]. 

We observed that the novel sequence from pompano has the closest similarity to a bacterium found on another host belonging to the family Carangidae [16]; therefore, it seems that closely related agents are found on closely related hosts. Unfortunately, the low intensity of infection could not allow extraction of an adequate quantity of DNA to attempt whole genome sequencing of the putative novel agent. Such material would have been useful for comparative genomics and would offer better insights into the phylogenetic relationships between these pathogens.

**Figure 4:**
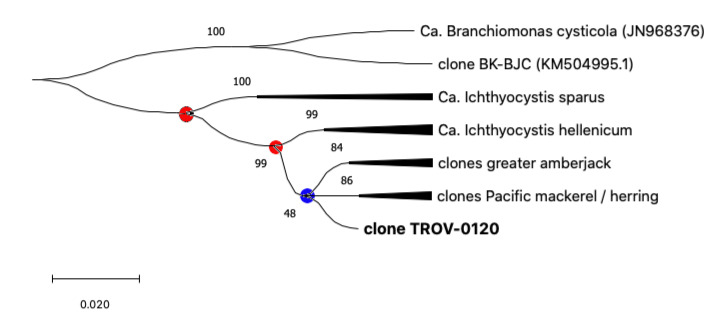
Phylogeny tree of the *Ca*. Ichthyocystis genus. All bacterial sequences from the same host are condensed into single branches to highlight evolutionary events leading to speciation (dots). Events that lead to the speciation of bacteria in gilthead seabream are highlighted with red dots, while the blue dot is used to indicate differentiation in other hosts.

Sequences from pompano, greater amberjack, Pacific mackerel, and Pacific herring share a single closer common ancestor with *Ca*. Ichthyocystis hellenicum and a more distant relative with *Ca*. Ichthyocystis sparus (Figure 3 and Figure 4). It might appear that the speciation event occurring between *Ca*. Ichthyocystis sparus and *Ca*. Ichthyocystis hellenicum emerged prior to the one parting *Ca*. Ichthyocystis hellenicum from agents in other hosts. The analysis of the partial 16S rRNA genes shows, therefore, that two different consecutive events lead to the speciation of bacteria in gilthead seabream (Figure 4, red dots) before epitheliocystis agents differentiated in other hosts (Figure 4, blue dot). This finding highlights the possibility that a beta-proteobacterium of the *Ca*. Ichthyocystis genus in *Sparus aurata* is an ancestor of the agents found in pompano, greater amberjack, Pacific mackerel, and Pacific herring.

Intensive aquaculture conditions, including the high density of hosts and stress, are believed to promote epitheliocystis intensity and disease development [1,24,29]. Gilthead seabream is one of the first fish species traditionally cultivated in the Mediterranean, initially extensively in coastal lagoons and saltwater ponds, and afterward (from the 1980s) intensively in seawater cages in the Mediterranean and coastal regions of the European Atlantic Ocean [30,31]. Of the other hosts related to *Ca*. Ichthyocystis agents in this study, only the greater amberjack has been recently cultivated, mostly in Spain, Greece, Malta, and Italy [32]. 

Potential roles of gilthead seabream in the evolution of the genus *Ca*. Ichthyocystis might be explored in the future by screening other wild and farmed sparids, while the impact of aquaculture on speciation dynamics might be discussed if other epitheliocystis agents from intensively reared species, such as the European seabass (*Dicentrarchus labrax*), are identified in early branches of the clade. Interestingly, epitheliocystis hyper infections have been reported in European seabass [33]. Crespo and colleagues in 2001 [33] suggest morphological similarity of the infectious agent in European seabass with the ones observed in gilthead seabream [34] and greater amberjack [35], currently associated with the *Ca*. Ichthyocystis genus. 

The current study, as well as the one on seabream [6] and a second one on greater amberjack [16], were all conducted in Greece; consequently, it might appear that the *Ca*. Ichthyocystis genus is especially present in the Eastern Mediterranean. On the other hand, to our understanding, most studies investigating the nature of epitheliocystis (or generally assessing gill health) in fish do not screen for this genus, potentially leading to an underestimation of the number of host species and geographic areas affected.

The presence of *Ca*. Ichthyocystis and epitheliocystis in natural populations of herring and mackerel must be verified. The NCBI-deposited sequences obtained from these fishes were produced during a study investigating bacterial diversity in dietary fish used to feed marine mammals in San Diego Bay of California and were not focused on epitheliocystis [36]. Bik and colleagues did not use gills but whole homogenized fish in their study; therefore, these sequences might not be associated with lesions. Moreover, since the aforementioned study was focused on mammals, the origin of the dietary fish from which the sequences were produced was not specified. Considering that the Pacific mackerel is not a Mediterranean species [37], confirming the presence of these agents in this host might lead to the expansion of the *Ca*. Ichthyocystis genus in other geographic regions.

## 4. Conclusions

This study extends the list of the beta-proteobacteria associated with epitheliocystis with the addition of a putative novel species of the genus *Ca*. Ichthyocystis. The genus currently comprises pathogens found in Sparidae, Carangidae, and potentially also Scombridae and Clupeidae.

Further screening of fish gills with *Ca*. Ichthyocystis-specific primers might be required to better appreciate the full extent of the diversity of these agents, their phylogenetic relationship, and attempt to understand the factors driving their evolution.

## Data Availability

All data have been provided in the paper.
